# Reporting outcome comparisons by sex in oncology clinical trials

**DOI:** 10.1038/s41467-024-47321-5

**Published:** 2024-04-09

**Authors:** Guo Zhao, Yuning Wang, Shuhang Wang, Ning Li

**Affiliations:** Clinical Trial Center, National Cancer Center/National Clinical Research Center for Cancer/Cancer Hospital, Chinese Academy of Medical Sciences and Peking Union Medical College, 100021 Beijing, China

**Keywords:** Outcomes research, Cancer therapy

## Abstract

Many aspects of human health and disease are influenced by sex as a biological variable and gender as a social construct. A recent study from *Nature Communications* reported the landscape of outcome comparisions by sex in oncology clinical trials, highlighting the need for a more thorough reporting of sex differences.

## The importance of sex differences in clinical research

The underlying basis for sex differences in health and disease can be attributed to sex chromosomes, with females typically having XX chromosomes and males having XY chromosomes in most mammalian species. Genes located on these chromosomes play crucial roles in the development of reproductive organs and production of sex steroids^1^. The term “sex” refers to the biological components based on sex chromosomal differences, including cellular and molecular differences^2^. “Gender” includes a series of factors, such as behavioural, environmental, and social factors and choices, that are related with an individual’s physiological and biological health^3^. Where biological factors are the main focus, comparisons between females and males in medical studies use the term “sex differences” rather than “gender differences”^4^.

Sex differences can be observed in various disease states in terms of the prevalence, diagnosis, prognosis, and therapeutic response. Females have distinct physiological characteristics that influence disease development and therapeutic responses to various interventions, which often diverge significantly from males^3^. Notably, in oncology practice, one significant difference between males and females is chemotherapy toxicity, with available evidence indicating that females exhibit greater susceptibility to the toxicity of various medications than males^5^. Despite the significant impact of sex differences on disease risk and treatment response, clinical decision-making often fails to consider a patient’s sex. This perspective can be partially attributed to historical unconscious biases, which reflects the fact that medical studies are mainly dominated by men^6^.

Fortunately, the awareness of sex differences and biases in academic and scientific communities has been growing. This is evidenced by the establishment of organisations like the Organisation for the Study of Sex Differences and the launch of the academic journal, Biology of Sex Differences. Additionally, recent regulations from the National Institutes of Health (NIH), such as The Sex and Gender Equity in Research (SAGER) guidelines^7^, mandate that grant applicants and clinical trials consider sex as a variable in biomedical studies^8^. In 2002, the World Health Organisation issued a gender policy that was subsequently adopted by the European Commission Directorate-General for Research and Innovation in 2003 and emphasizes the importance of incorporating women in medical research^2^. Currently, it is required that women be included in clinical trials, with pregnancy testing being a component of the inclusion criteria^9^. However, these policies are not widely followed, information on sex is often not collected, and comparisons between male and female participants in trials are often not provided in the medical literature^10^.

The recommendation made by regulatory agencies to ensure women are included in clinical researches and sex differences are adequately analysed warrants further consideration within the precision oncology community. The development of sex-specific personalised treatment has the potential to benefit both male and female patients. Therefore, there is an urgent need for a comprehensive study that assesses the current status of sex differences in oncology clinical trials and recommends the most appropriate future directions for precision oncology.

## Current landscape of outcome comparisons by sex in oncology clinical trials

A recent data analysis article reported by Kammula et al. in Nature Communications^11^ represents a significant response to this urgent research need, which will be studied and applied by oncology field for years to come. This study comprehensively characterised sex outcome comparisons in all oncology interventional clinical trials and identified comparisons that found a significant difference between males and females. Leveraging the paid service called Trialtrove® (https://clinicalintelligence.citeline.com/), Kammula et al. asked several questions, including: What types of evidence for sex differences are reported and how often are they disclosed in oncology clinical trials; can significant outcome differences by sex be observed in certain cancer types, treatments, and measurements; and how can these findings translate into future directions that increase adequate sex comparisons in oncology clinical trials?

This study used a series of reliable methods to ensure that almost all oncological interventional clinical trials worldwide were included. Trialtrove® is an online database that collects and curates data from ClinicalTrials.gov and thousands of other sources in a semi-structured format. The use of Trialtrove® to collect the data of clinical trial have been widely accepted and used. Due to the daily update of Trialtrove®, a single data freeze (in December 2022) was set to have a stable set of data to further analyse and all data of 89,221 oncology trials was downloaded and initial filtered based strict inclusion and exclusion criteria. After the initial filtering, a series of Python methods and codes were applied to identify trials with sex comparisons. Finally, potential results were manually curated based on Trialtrove annotations and original papers. Through a series of rigorous curation and quality control measures, this study has a much broader scope than prior research, and 472 trials were found to report at least one sex comparison between males and females.

Kammula et al. provided a plethora of results, with the following key findings: direct statistical comparisons by sex in outcomes or side effects in papers that report oncology clinical trial results are rare, accounting for only 0.5% of all 89,211 trials; females generally exhibit better survival outcomes and treatment responses than males in the majority of clinical trials; and males have fewer side effects than females. However, this survival advantage in females is mainly driven by trials for non-small cell lung cancer (NSCLC), non-Hodgkin’s lymphoma (NHL), and acute myeloid leukaemia (AML), rather than across all cancer types. Sex-specific differences are observed in the efficacy of certain treatments, including epidermal growth factor receptor (EGFR) inhibitors in NSCLC and rituximab (a CD20 monoantibody) in NHL, which go beyond the general disparities in survival between sexes in these two cancers.

## Conclusions and recommendations

**Fig. 1:**
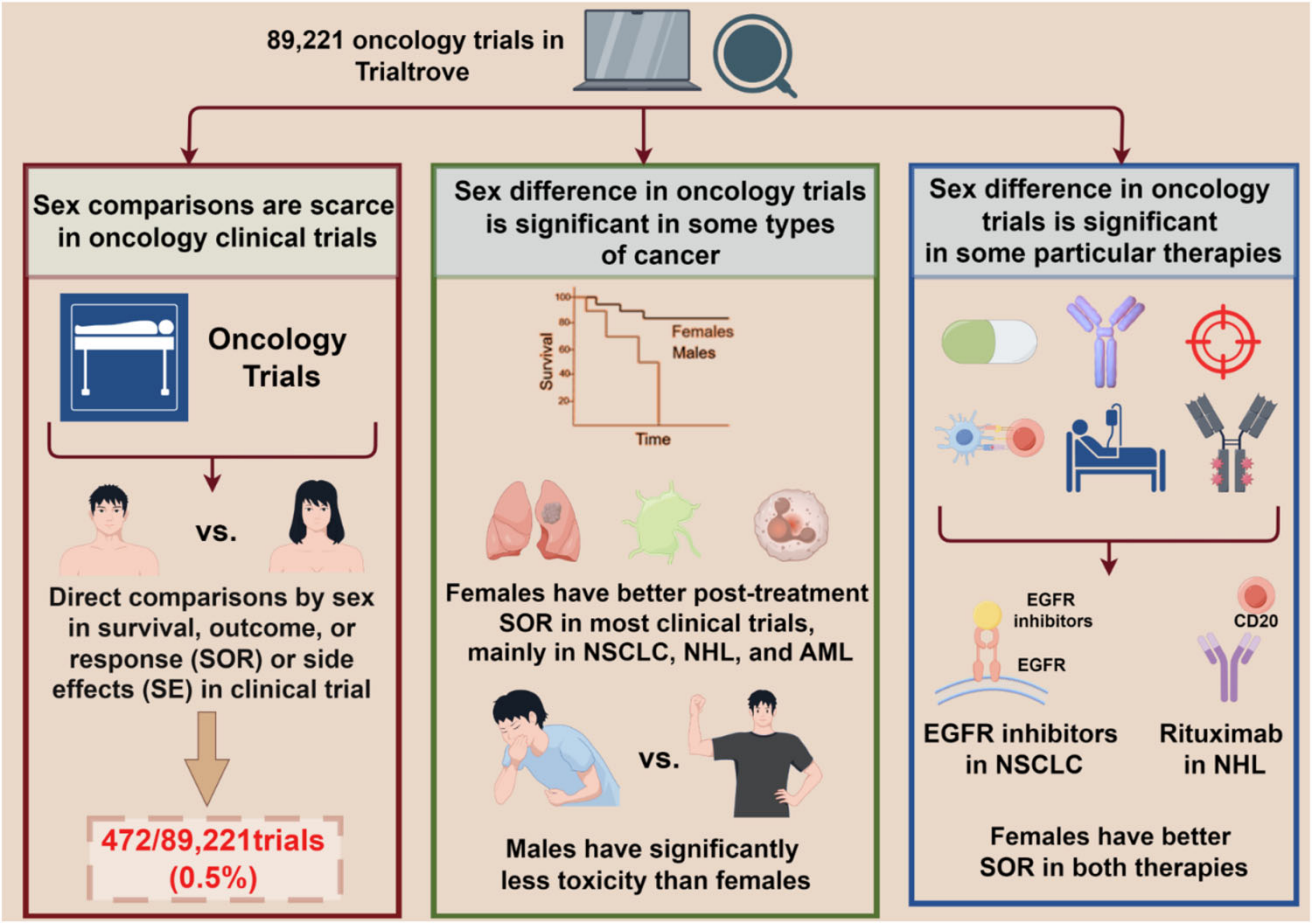
Outcome differences by sex in oncology clinical trials by Trialove®. First, direct statistical comparisons by sex in outcomes or side effects in oncology clinical trials results papers are rare, only accounting for 0.5% of all 89,211 trials. Second, females generally exhibit better survival outcomes and treatment responses than males in the majority of clinical trials whereas males have fewer side effects than females. However, this survival advantage of females is mainly driven by trials for NSCLC, NHL, and AML. Third, sex-specific differences are observed in the efficacy of certain treatments, including EGFR inhibitors in NSCLC and rituximab in NHL.

Overall, the findings of Kammula et al. present a comprehensive and up-to-date landscape of outcome comparisons by sex in oncology clinical trials. This study found that only 472/89,221 oncology clinical trials (0.5%) curated post-treatment sex comparisons. Furthermore, 42% of trials have indicated that females have better survival, outcome, or response (SOR), while only 16% reported that males have significantly better SOR. In addition, males have presented fewer side effects than females in clinical trials, and two treatment/cancer type combinations drive favourable SOR outcomes in females: EGFR inhibitors in NSCLC and rituximab in NHL (Fig. 1).

Due to the expansive interpretation of the 1977 US Food and Drug Administration (FDA) guidelines, which recommended the exclusion of women of childbearing potential from participating in early phase clinical trials owing to inadequate safety data, women have been predominantly omitted from clinical research studies for decades^8^. Since the implementation of the 1993 FDA guidelines that recommended the inclusion of women in early phase trials and advocate for the essential analysis of sex differences, there has been an increase in the enrolment of women. However, a predominant proportion of participants in early phase trials continued to be men^12^. In 2014, the FDA again highlighted the critical importance of “improving completeness/quality of demographic subgroup data collection, reporting, and analysis, and making demographic subgroup data more available and transparent^8^.”

This study shows the impact of and room for improvement in the current policies for identifying sex-specific results in oncology clinical trials. Reports of sex differences in oncology clinical trials are rare and indicate the urgent need for related laws and guidelines in the field of oncology. Oncology clinical trials are required to test the safety and effectiveness of promising treatments in patients^13^. Although differences in chemotherapy-related toxicity between males and females have been observed for several decades, a few key questions remain: do male patients show better outcomes in response to immunotherapy, and do females generally have better survival in oncology clinical trials? These are hot topics in the field of oncology, and more data on sex comparisons between males and females from clinical trials will help build a solid foundation for exploring these issues.

Sex is an important factor in oncology research, and further studies are required to elucidate the direct sex differences between males and females. The following recommendations for improving the reporting sex differences in oncology clinical trials may improve our understanding and comparability of findings across studies, and help achieve true precision oncology. (1) The NIH and FDA should reach a consensus on the regulatory guidelines of sex-specific comparisons in oncology clinical trials; (2) top-tier scientific and oncology-specific journals and publishers should establish more transparency and rigorous guidelines for reporting sex differences; (3) clinical trialists and biostatisticians should direct how ideal trials are conducted with gender diversity in mind by considering data collection and analysis while planning the study; and (4) medical researchers and doctors should provide comprehensive data disclosure to enable themselves and others to conduct the essential analysis of sex differences. Only with the collaborative efforts from all scientific communities will it be possible for oncology to move towards sex-specific personalised oncology.
